# Citrus Pomace Biomass as a Source of Pectin and Lignocellulose Fibers: From Waste to Upgraded Biocomposites for Mulching Applications

**DOI:** 10.3390/polym13081280

**Published:** 2021-04-14

**Authors:** Domenico Zannini, Giovanni Dal Poggetto, Mario Malinconico, Gabriella Santagata, Barbara Immirzi

**Affiliations:** Institute of Polymers, Composites and Biomaterials, National Research Council, Via Campi Flegrei 34, 80078 Pozzuoli, Italy; domenico.zannini@ipcb.cnr.it (D.Z.); giovanni.dalpoggetto@ipcb.cnr.it (G.D.P.); mario.malinconico@ipcb.cnr.it (M.M.); gabriella.santagata@ipcb.cnr.it (G.S.)

**Keywords:** citrus pomace waste, pectin, circular economy approach, green extraction, biocomposites, mulching application

## Abstract

Citrus pomace derived from the industrial processing of juice and essential oils mostly consists of pectin, cellulose, hemicellulose, and simple sugars. In this work, citrus pomace waste from an agricultural company in South Italy was used as source of pectin. The extraction conditions of the polysaccharide were optimized using a suitable combination of time and a concentration of a mild organic solvent, such as acetic acid; thus recovering high M_w_ pectin and bioactive molecules (flavonoids and polyphenols). The pectin was structurally (GPC, FTIR), morphologically (SEM), thermally (TGA/DTG), and mechanically characterized, while bioactive molecules were separated and the total phenolic content (TPC) and total flavonoids content (TFC) were evaluated. With the aim to develop novel biocomposite-based materials, the pectin extracted from citrus waste was reinforced with different amounts of lignocellulose fractions also recovered from citrus waste after polysaccharide extraction, according to a “zero waste” circular economy approach. The prepared biocomposites were morphologically and mechanically characterized to be used as biodegradable mulching systems for crop protection. Thus, the citrus waste biomass was recovered, fractionated into its main raw materials, and these were recombined to develop novel upgraded biocomposites for mulching applications, by means of a cost-effective and eco-sustainable approach.

## 1. Introduction

Citrus is the one of the largest and most popular commercially grown fruit crops across the world. Annually, it is estimated that 33% of the total global citrus harvest is used for juice and essential oil production [1]. In Italy alone, about 3 million tons of citrus fruit are produced annually: mainly lemons, oranges, bergamot, and clementine. The citrus processing industries produce huge amounts of waste: over 40 million tons worldwide, which plays a key role in global environment impact, since the huge organic and water load of landfilled biomass leads to the formation of greenhouse gases and microbial contamination during bulk decomposition [2]. A valid eco-sustainable alternative to citrus waste landfilling is the exploitation of the biomass as a source of high added value compounds and macromolecules [3]. Actually, there is an emerging interest in using agricultural and food processing waste as a source of bioactive molecules for functional food products or as active pharmaceutical compounds [4]. It is suffice it to say that the reuse of waste or by-products in the food industry can increase economic value, provide environmental benefits, and better highlight eco-sustainability [5,6,7].

All the transformation processes used in the citrus industry give rise to three main products: juice, essential oil, and the residual pulp fraction, whose percentages are approximately 35–45%, 0.2–0.5%, and 55–65%, respectively. Actually, more than a waste, the pulp recovered after juice and oil extraction can be considered a processing by-product that is a source of active compounds, such as flavonoids and d-limonene, macromolecules, such as pectins and simple sugars, lignocellulose peel fibers, seed oils, and ethyl alcohol, which can be reintegrated and reused as secondary upgraded raw materials in the agro-food production chain [8,9,10,11,12,13].

Among them, pectin represents 45% of the total citrus waste by weight, together with soluble sugar, cellulose, and hemicellulose [14].

Pectin is a natural constituent of all terrestrial plants that is particularly abundant in the primary cell walls of fruit and vegetable. The chemical composition and structure of pectin is very complex and depends on the source, extraction methods, plants, storage, and maturity of the raw plant source. Nevertheless, it is generally accepted that the pectin structure consists of heterogeneous polysaccharides with three main structural domains covalently linked one to another, the homogalacturonan, and the highly branched rhamnogalacturonans regions [15,16,17,18,19,20,21,22]. Pectins are categorized according to their degree of esterification or methoxylation (DM), i.e., the percentage of carboxyl groups esterified with methanol. Pectins with a DM > 50% are high-methoxyl pectins (HMP); those with a DM < 50% are low-methoxyl pectins (LMP) [23]. For instance, high-methoxyl pectins form a gel when they are heated in low pH (2–3.5) water solutions containing a high concentration of sugars (about 55–75%). Low-methoxy pectins form partly shear reversible gels in the presence of calcium ions. Hence, the esterification degree and the structural features of pectin, like molecular weight, are responsible for the functional properties of pectin [24,25]. Commercially, both citrus and apple pectins are often used as preservative, gelling, and thickening agents in the food processing industry because of their high DM and molecular weight [26]. The gelling, binding, biocompability, and nontoxic properties of pectin make it a promising biopolymer for biomedical and pharmaceutical applications [27].

Frequently, pectin is extracted from citrus peels and apple pomace using the acid extraction technique. Acids are highly effective at solubilizing and stabilizing the pectin that is tightly bound in the cell wall, making them the most effective extracting agents. Typically, industrial pectin production is implemented using acidified water with hydrochloric, nitric, sulphuric acid, or organic acids at pH 1.0–3.0, high temperatures (70–90 °C), and extraction times of 1–12 h, depending on the desired properties of the pectin [28].

However, it is widely reported that the use of strong acids could result in pectin depolymerization and de-esterification, as well as disposal and environmental issues due to their corrosive and toxic nature [29]. Therefore, the experimental extraction conditions (temperature, extraction acid, time, and pH) should be carefully controlled to achieve the desired pectin quality and yield [30].

In this paper, pectin was extracted from citrus pomace biomass using low concentrations of a mild organic acid, such as acetic acid. This choice was driven by the intended agricultural application of pectin. In fact, the author partnership has a long-standing expertise in developing biodegradable mulching films and biocomposites based on the water solutions of polysaccharides and cellulose fibers. The solutions are directly sprayable onto the soil and are able to form a protective geomembrane against the growth of spontaneous weeds that affect crop production. At the end of the cultivation period, the geomembranes can be buried into the soil where they are biodegraded by bacterial flora. As an example, sodium alginate water solutions, glycerol as a plasticizer, and cellulose fibers as a reinforcing agent have been used as mulching materials for strawberry crop production under greenhouse conditions [31]. Another mulching application concerns the use of chitosan as a biopolymer matrix dissolved in an acetic acid solution at 3% *v/v*. In fact, acetic acid is an admitted substance in agronomic practices, being widely used as a natural herbicide [32]. Moreover, chitosan has been reported to possess antifungal and antibacterial activity and it has been shown to be effective in soil amendment, providing many benefits to different plant species by reducing pathogen attacks and infection [33]. The use of chitosan as a protective mulching geomembrane was demonstrated by the authors. It can form water stable films on the soil that are able to protect the cultivation crops from spontaneous weeds growth by exploiting local and systemic crop defense responses [34,35]. In their previous studies, the authors found that the use of 3% *v/v* of acetic acid in water solution represented a good compromise to perfectly dissolve chitosan without compromising its depolymerization.

In this paper, the same concentration of acetic acid was used to extract pectin from citrus waste biomass and then redissolve it so it could be sprayed on the soil. With the aim of reinforcing the geomembrane, lignocellulose fractions recovered from the same citrus biomass after pectin extraction were added. As far as we know, there is no literature data concerning the development of biocomposites based systems in which both the polymer and fibrous dispersed phase are derived from the same biomass. Specifically, from the pulp, pectin was extracted and separated from the solid fraction represented by fibers; pectin and fibers were then recombined in functional biocomposites. This novel approach could be framed in the context of the circular economy, as it provides the recovery of biomass, separation into its components, and their subsequent recombination in an upgraded form of secondary raw materials; thus, achieving an environmentally sustainable and low-cost closure following the zero-waste circular economy route [36]. In this paper, high molecular weight pectin was extracted in mild conditions from citrus waste biomass. The biopolymer was structurally (Gel Permeation Chromatography, GPC), (Fourier Transform Infrared, FTIR), morphologically (Scanning Electron Microscopy, SEM), thermally (Thermal Gravimetrical Analysis/Derivative Thermogravimetry, TGA/DTG), and mechanically characterized; from the citrus pulp, bioactive molecules were separated and the total phenolic content (TPC) and total flavonoids content (TFC) were evaluated. With the aim to develop novel biocomposite-based materials to be sprayed on the soil as a mulching geomembrane, the fiber residues obtained after pectin and biomolecule extraction were recovered and added to the pectin solutions. Preliminary morphological and mechanical characterizations of the biocomposites were performed in order to evaluate their potentiality as biodegradable mulching films.

## 2. Materials and Methods

### 2.1. Material and Chemicals

Citrus waste was supplied by Università Mediterranea of Reggio Calabria (Reggio Calabria, Italy), with a moisture content of about 15% by weight. Analytical-grade glacial acetic acid (HAc), isopropanol, ethanol, acetone, bicarbonate sodium (NaHCO_3_) Folin–Ciocalteau reagent, aluminum trichloride (AlCl_3_), sodium nitrite (NaNO_2_), sodium hydroxide (NaOH), and hydrochloric acid (HCl, 36%) were purchase from Sigma-Aldrich, Merck Group (Saint Louis, MI, USA). Citrus Pectin Classic CU 701 (PEC) was kindly supplied by Herbstreit & Fox (Neuenbürg, Germany), with a degree of esterification of 34% and a galacturonic acid content of 86%.

### 2.2. Extraction and Purification of Pectin from Citrus Waste

In a typical extraction, 20 g of powder citrus waste were dispersed in 200 mL of a solution of 3% (*v/v*) HAc (pH = 2.6). The pectin extraction was performed at 90–100 °C for 6 h under magnetic stirrer. Afterwards, the suspension obtained was rapidly cooled to room temperature, and filtered to separate the liquid extract from the insoluble fraction. In order to definitively remove the solid residues, the liquid extract was centrifuged for 40–50 min at 10,000× *g* at 4 °C.

Once the liquid fraction was recovered, the pectin was precipitated with an excess of isopropanol (a twice the volume with respect to the liquid extract volume) and filtrated on paper filter.

The pectin was washed with ethanol and put into a vacuum oven at 60 °C until reaching a constant weight. Finally, the percentage yield of the pectin was determined through the following equation (Equation (1)):

(1) $$\mathrm{Yield}\;(\%)=\left(\frac{a}{b}\right)\times 100$$

where *a* corresponds to the quantity of pectin obtained from the extraction, and *b* corresponds to the initial amount of powder citrus waste [37,38]. The percentage yield of the extracted pectin was around 24%.

The alcoholic fractions of isopropanol and ethanol were mixed and evaporated using a rotavapor instrument; the obtained powder was analyzed to evaluate the total phenol (TPC) and flavonoid contents (TFC).

The lignocellulose fraction, recovered after the pectin extraction, was washed with a NaHCO_3_ (3% wt.) solution until neutrality and dried under vacuum. Finally, it was ground through a mill and sieved then recombined with the pectin for the preparation of the biocomposites.

### 2.3. Pectin Film and Biocomposites

The amount of the dried pectin recovered from 10 mL of the liquid extract was about 0.30 g. Firstly, a film of purified pectin (Purified PEC), used as control, was prepared by dissolving 1.00 g of pectin in 35 mL of 3% *v/v* of acetic acid water solution. The solution was poured in a Petri dish and allowed to dry at room temperature under a ventilated hood. In order to follow a cost-effective approach, the other films of pectin were prepared by casting 35 mL of liquid extract containing about 1.00 g of pectin. In order to follow a holistic approach by recombining the pectin and the fiber residual fraction to obtain biocomposites, 0.15 g, 0.25 g, and 0.35 g of lignocellulose fibers, recovered after pectin extraction, were added to the polymer matrix and casted on Petri dish and dried as previously described. Thus, biocomposites, named PEC-15, PEC-25, PEC-35, respectively, were obtained.

The film and biocomposite compositions and their identification codes are reported in Table 1.

### 2.4. Characterizations Methods

#### 2.4.1. Gel Permeation Chromatography (GPC)

Gel permeation chromatography was performed on pectin extracted at different times or acidic contents. The analysis was carried out using a GPC Max Viscotek (Malvern, UK) Phenomenex (Torrance, CA, USA) system equipped with a TDA 305 detector (Refractive Index, Low Angle Light Scattering, Right Angle Light Scattering and Viscometer) and UV detector. We used a pre-column TSK PW_XL_ and TSK Gel GMPWXL. All the samples were dissolved at 2 mg/mL concentration and eluted in MilliQ water containing 0.2% NaN_3_ and 0.1 M NaNO_3_. After complete dissolution, samples were filtered through membranes of 0.22 μm porosity. The injection volume was 100 μL, and the flow rate was 0.6 mL/min. The chosen method of analysis was triple point, calibrated with a PEO standard, provided by Viscotek, with a narrow molecular weight distribution. The measurements, performed at 40 °C according to the temperatures of the columns and detectors, ran for 60 min in triplicate.

#### 2.4.2. Degree of Methoxylation (DM) Measurement

The DM value of extracted pectins was obtained using a titrimetric method [39]. First, 2 mL of ethanol was added to the 200 mg sample with 20 mL of distilled water. It was mixed thoroughly until complete dissolution. Two drops of phenolphthalein were added to the mixture and the mixture was titrated with NaOH solution (0.1 M) until it appeared pale pink. The volume of added NaOH after the titration was noted as V_1_. Afterward, the solution was mixed with 10 mL of NaOH. The mixture was shaken for 1 h at room temperature. Then, 10 mL of HCl solution (0.1 M) was added and the mixture was stirred until the pink color disappeared. The solution was titrated again with NaOH solution (V_2_). The DM value of pectins was calculated according to the following equation (Equation (2)), all the measurements were performed in triplicate, and the results are reported as averaged values.

DM (%) = [V_2_/(V_2_+V_1_)] × 100 (2)

#### 2.4.3. Attenuated Total Reflection Fourier Transform Infrared Spectroscopy (FTIR-ATR)

FTIR analysis was recorded in an Attenuated Total Reflection near FTIR-ATR (ATR modality) on powder pectins using a Perkin-Elmer Spectrum 100 (Waltham, MA, USA). The samples were dried under vacuum for 24 h at 60 °C to remove water traces. Spectra were recorded as an average of 16 scans (range: 4000–650 cm^−1^, resolution: 4 cm^−1^).

#### 2.4.4. Total Phenolic Content (TPC)

Determination of the TPC was performed according to [40]. Briefly, 125 μL of sample solution (2% *w/v* dry mass of alcoholic liquid fraction in H_2_O) were mixed with 125 μL of Folin–Ciocalteau reagent. After 6 min of incubation, 1.25 mL of aqueous solution 7% *w/v* NaHCO_3_ were added and the final solution was left at room temperature for 90 min in the dark. After this time, the absorbance was measured at 760 nm using a UV-Vis spectrophotometer (V−570 Jasco, double beam system with a single monochromator, Easton, Van Nuys, LA, USA). To calculate TCP, the calibration curve of catechin (0–600 μg) was calculated. The result, in duplicate, was expressed as mg of catechin equivalents (CE) per g of sample dry weight (mg CE/g).

#### 2.4.5. Total Flavonoids Content (TFC)

TFC was evaluated according to [41]. A sample solution of 0.5 mL (2% *w/v* dry mass of alcoholic liquid fraction in H_2_O) was mixed with 2 mL of H_2_O and 0.15 mL of 5% *w/v* NaNO_2_ and final solution was incubated at room temperature for 6 min. Then, 0.15 mL of 10% *w/v* AlCl_3_ were added and left at room temperature for 6 min. Finally, 2 mL of 4% *w/v* NaOH and 0.7 mL of H_2_O were added and the mixture was left at room temperature for supplementary 15 min. The absorbance was measured at 510 nm using a UV-Vis spectrophotometer (V−570 Jasco, double beam system with a single monochromator, Easton, Van Nuys, LA, USA), the calibration curve was obtained from the known concentrations of catechin (0–600 μg), and the TFC was expressed as mg of catechin equivalents per g of sample dry weight (mg CE/g). Measurements were performed in duplicate.

#### 2.4.6. Thermogravimetric Analysis (TGA)

Thermogravimetrical analysis of pectins and lignocellulose residue were performed using a thermogravimetric analyzer TGA/DTG Perkin-Elmer PyrisDiamond, equipped with gas station. A 3–4 mg sample was placed in an open ceramic crucible and heated from 25 °C to 600 °C at a speed rate of 10 °C/min, under nitrogen at 30 mL/min. Before testing, samples were kept under vacuum at 60 °C for 24 h.

#### 2.4.7. Mechanical Tests: Tensile Test and Puncture Test

In order to simulate the natural pressure exerted by plants against the films, two different mechanical tests were performed: the tensile test and the puncture test, which is an empiric test previously used by [31]. All the mechanical analyses were performed on six specimens, previously conditioned at room temperature and at air relative humidity equal to 50%. Tensile tests were carried out by a dynamometer model 4301, Instron (Canton, OH, USA) equipped with a load cell of 1 kN. The measurements were performed on dumbbell-shaped films. The width and the length of investigated films were 4 and 28 mm, respectively, while the thickness of each film was measured using a digital micrometer (IP65 Mitutoyo, Kawasaki, Japan). The values were reported as the mean value of three different points measurements as the average value (0.1 ± 0.3 mm). All the measurements were carried out at 25 °C and 50% relative humidity (RH), at a crosshead rate of 2 mm/min. Young’s modulus, stress, and strain at break points were determined. The puncture test consisted in penetrating the specimens until the film was lacerated [31,42]. More specifically, the samples to be tested, opportunely cut with a 40 mm circular-section punch cutter, were trapped in cups fixed on the inferior traverse of the INSTRON instrument. They underwent the action of a force exerted by a spherical dart linked to a steel rod fixed on the upper traverse of the apparatus; the dart, moving down at a fixed rate of 2 mm/min, penetrated the sample until the rupture of the film. The applied load as a function of the displacement was recorded; the parameters obtained were normalized with respect to 1/2 of the area of the sphere and with respect to an estimated value of the displacement of 10 mm.

#### 2.4.8. Scanning Electron Microscope (SEM)

Morphological analysis of powder pectins, film, and biocomposites were performed by means of a scanning electron microscope (SEM) (Quanta 200 FEG, 338 FEI, Eindhoven, The Netherlands), on powder samples. Surfaces were coated with a homogeneous layer (18 ± 0.2 nm) of Au and Pd alloy by means of a sputtering device (MED 020, Bal-Tec AG, Tucson, AZ, USA). The micrographs were performed at room temperature, in high vacuum mode. By means of energy dispersive X-ray spectroscopy (EDS), it was possible to perform the chemical analysis of selected microscopic regions. EDS was performed in the SEM by means of an Oxford Inca Energy 250 System equipped with an INCAx-act LN2-free detector, using an accelerating voltage of 20.0 kV.

## 3. Results

Typically, industrial pectin production is performed by means of acid extraction methods, using hydrochloric, nitric, sulphuric acid, or organic acids at pH 1.0–3.0, high temperatures (70–90 °C), and extraction times of 1–12 h, depending on the desired properties of the pectin. However, the use of strong acids can induce pectin depolymerization, which affects its final properties.

In this paper, the use of acetic acid could allow pectin extraction from citrus waste without affecting its structural and functional properties. Several concentrations of acid and different extraction times were evaluated in order to reach the best compromise of processing parameters and pectin properties. On the basis of previous analyses performed by the authors on other pectin sources (results of which are not the subject of this manuscript), preliminary experiments on citrus biomass were performed using a high concentration of acetic acid solutions, specifically 9% *v/v* of acetic acid for 2, 4, and 6 h. As a result, the extraction yield increased: from 16.4% after 2 h to 23.7% after 6 h of treatment. However, the methoxylation degree decreased [43]. In literature, it is reported that the demethoxylation process mainly occurs at basic pH [44]. However, Fraeye et al. [45] demonstrated that for a pH lower than 3, DM decreases due to acid catalyzed demethoxylation. In the tested samples, the high concentration of HAc (PEC 9%HAc) combined with the high extraction temperature and prolonged times synergically acted to decrease the DM. For this reason, the acid concentration was reduced to 3% *v/v*. The choice was suggested from the application point of view. As previously stated, the authors have corroborated experience in mulching geomembrane development using polysaccharide water solutions containing 3% *v/v* of acetic acid [31]. The lower concentration of acid prevented the polysaccharide dissolution, while higher concentrations of acid negatively affected the results of the tests, since the acetic acid did not provide a suitable selective action as a herbicide; as a result, the crop growth and quality were seriously impaired (data will be published in an upcoming paper in collaboration with an Italian agronomic partnership). Effectively, according to the results reported in Table 2, the use of a lower concentration of acid and an average time of 6 h produced suitable, efficient, and more interesting values for both yield and DM.

### 3.1. Gel Permeation Chromatography (GPC)

Pectin samples were analyzed through gel permeation chromatography to investigate molecular weights and differences in extraction conditions. For all purified samples, an evaluation of dn/dc: a characteristic value for a sample–solvent–temperature combination, was performed in triplicate.

**Figure 1:**
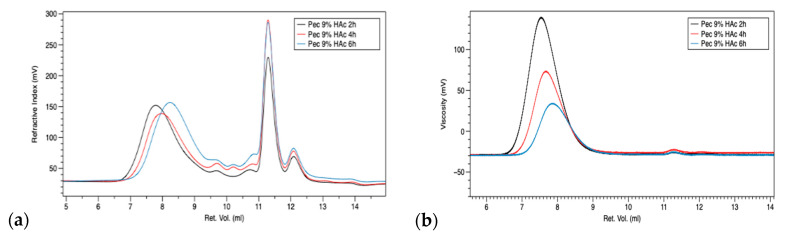
Refractive index (**a**) and viscometer (**b**) chromatograms of pectins at 9% HAc and different extraction times: 2 h—black, 4 h—red, 6 h—blue.

Data reported in Table 3 highlight a clear decrease in M_w_, M_n_, and IV with the increasing extraction time with an acetic acid concentration of 9%, indicating that pH and extraction time play an important role in pectin structure and its final properties; however, pectin extracted with 3% HAc for 6 h shows higher values of all parameters. By observing chromatograms at a refractive index (RI) detector of 9% HAc series (Figure 1a), it is possible to note that the increase in extraction time (2–6 h) leads to degradation of higher molecular weights (differences in the starting point of the curves in the 6.7–9.5 mL region) and an increase in the area in the 9.5–12 mL region, corresponding to the lower molecular weights-oligomeric fraction, together with other fractions present in citrus waste, such as polyphenol, flavonoids, and others. This behavior is also supported by chromatograms analyzed through a viscometer detector (Figure 1b), in which the decrease in viscosity emphasizes the degradation trend.

**Figure 2:**
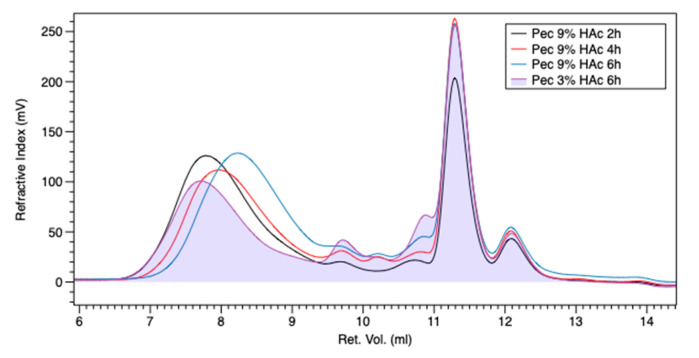
Refractive index detector chromatograms of pectins at 3–9%-HAc and different extraction times: 2 h—black, 4 h—red, 6 h—blue, and 3%-HAc-6 h—violet.

Comparing this trend with PEC-6h-3% (in Figure 2 it was indicated as PEC 3%-HAc-6 h), it is possible to observe that the polymeric peak is detected at a higher retention volume (higher molecular weights) for the PEC-2h-9% (in Figure 1 and Figure 2 it was indicated as PEC 9%-HAc-2 h) sample. Comparing the area of the PEC-6h-3% chromatogram in the 9.4–12 retention volume range with the corresponding area of PEC-2h-9%, we can note that in the last chromatogram, there is a big increase in a low molecular weight fraction, which accounts for the best mechanical performance of the pectin extracted with low HAc content. In fact, this low molecular weight fraction acts as a plasticizer in appropriate quantities to achieve the best mechanical properties.

### 3.2. Attenuated Total Reflectance Fourier Transform Infrared (FTIR-ATR) Spectroscopy

**Figure 3:**
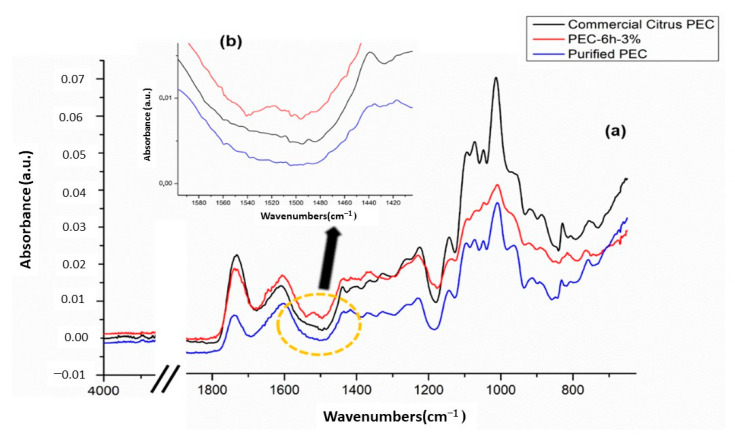
FTIR-ATR spectrum of all pectin samples (**a**) and the presence (**b**) of bioactive molecules peak.

In Figure 3a, we present the FTIR spectra of pectin recovered after 6 h—3% (*v/v*) of HAc, purified pectin, and a FTIR spectrum of commercial citrus pectin. In general, for all pectins, it is possible to observe that the strong and wide peak at 3364 cm^−1^ was attributed to the O–H elongation vibrations, and the 2936 cm^−1^ peak could be induced by the C–H elongation vibrations of the CH_2_ and CH_3_ groups in the pectin. The absorption peaks shown at 1731 cm^−1^ and 1608 cm^−1^ were assigned to the C=O stretch vibration of the methyl esterified carboxyl and to the asymmetric stretch vibration C=O of the free carboxyl, respectively. The absorption bands at 1143 cm^−1^ and 1048 cm^−1^ corresponded to the stretching vibrations of the lateral groups C–OH and to the vibration of the glyosidic bond C–O–C [46]. Figure 3a shows the FTIR-ATR spectra of the PEC-6h-3%. In the region 3400–2500 cm^−1^, a wide absorption band is observed because of the stretching vibrations caused by intermolecular interactions through the O–H bonds between the pectin monomers. The peak at around 2950 cm^−1^ refers to the C–H vibrations of the methynic groups in the polymer chain and the methyl group of the methyl ester. These include the bending and stretching vibrations of CH, CH_2_, and CH_3_ [47]. The peaks at 1760–1730 and 1630–1600 cm^−1^ were due to the relative contribution of the ester carbonyl groups (C=O) and to the stretching of the carboxylate ions (COO^−^), respectively [48]. The carboxylate groups show a weaker symmetrical stretch band near 1400 cm^−1^. In the pectin samples, the weakest COO^−^ symmetric stretch is followed by moderately intense absorptions between 1300 and 800 cm^−1^, collectively defined as the region of the fingerprint that is unique for a compound. These bands are generally difficult to interpret. Other minor bands in pectin samples are C–H bending and C–O stretching occurring at 1380 cm^−1^ and 1300–1000 cm^−1^, respectively [49]. The absorption peaks at 1100–1040 cm^−1^ were assigned to the C–O–C stretch vibrations of the polymer chain structure [50]. In particular, it is possibly evidenced that in the spectrum of PEC-6h-3%, a sharp absorption band is also observed between 1600 and 1500 cm^−1^ due to aromatic double bonds (aromatic rings) related to the presence of bioactive molecules (circled by the yellow circle), as indicated by the black arrow in Figure 3b. Moreover, at around 1520 cm^−1^, there is a new peak, indicating the functional group of the phenolic compound in the PEC-6h-3% sample [51]. This confirms that simply washing the liquid extract with alcohols does not completely remove the polyphenols fraction. Moreover, the spectrum of purified pectin obtained is perfectly comparable with commercial citrus pectin. Thus, the purification treatment recovers a pectin that is very similar to commercial pectin, as shown from Figure 3a.

On this basis, we performed the preliminarily evaluation of the fraction soluble in the hydro-alcoholic phase obtained during the purification of the PEC-6h-3% sample. The recovery of dry mass, TPC, and TFC was assessed as 46% w (with respect to the initial mass of the powder citrus waste), 46.34 ± 2 mg CE/g, and 34.42 mg ± 3 CE/g, respectively. Further studies are needed to investigate the antioxidant activity, composition, and other properties of pectin-enriched films for applications such as food-packaging smart materials with the aim of obtaining value-added products from bio-waste.

### 3.3. Thermogravimetric Analysis (TGA)

**Figure 4:**
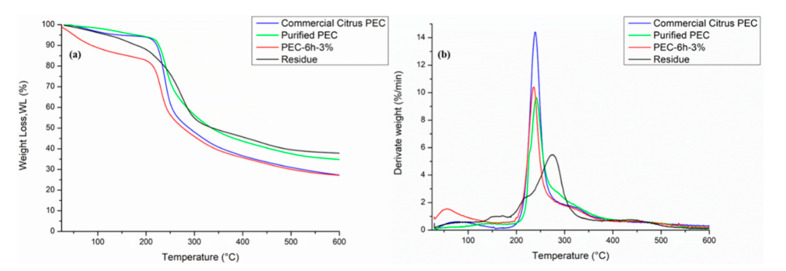
Thermogravimetric analysis (TGA) (**a**) and Derivative Thermogravimetry (DTG) (**b**) thermograms of all pectins and the lignocellulose residue solid.

In Figure 4a,b, we present the TGA thermograms and their Derivative Thermogravimetry (DTG) of commercial citrus pectin, PEC-6h-3%, and purified pectin. All curves were normalized with respect to the initial sample weights. The results of the thermogravimetric analyses are presented in Table 4. The TGA curve records the weight loss of pectin during heating. First of all, from Figure 4a,b, it is possible to observe that the thermal degradation pattern of purified pectin is similar to that of commercial citrus pectin. This confirms that the extracted pectin, which was subsequently purified in a step-by-step process, is comparable to commercial citrus pectin.

**Figure 5:**
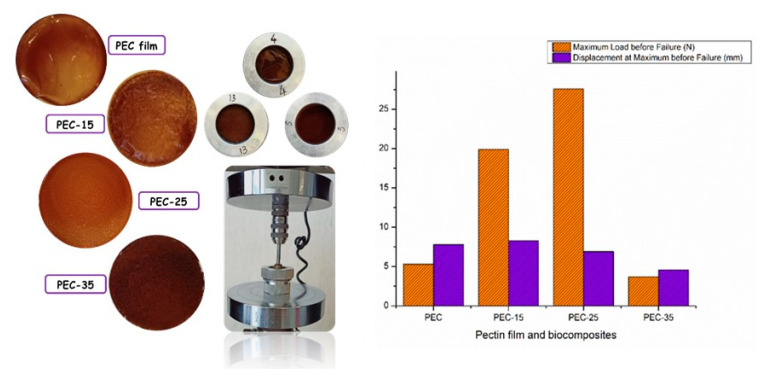
Puncture tests of the pectin film and biocomposites.

The value of the force to breaking of the biofilms increases from PEC to PEC-25, as can be seen from Figure 5. For the PEC-35 biocomposite, it is possible to observe a decrease in the value of the force to breaking. Evidently, the presence of filler in an elevated amount induced a poor interfacial adhesion between the liquid matrix and the residue, which is also jointly responsible for the scarce mechanical stress transfer from the continuous to the dispersed phase.

In our case, it was possible to observe that the thermogravimetric curves of the pectins (Figure 4a) principally presented two thermal steps: the first thermal step consisted of a weight loss of up to 10% in the 50–120 °C range, which was related to the evaporation of the water present in the sample; the second thermal step started around 200 °C. In this step, a rapid mass loss occurred, which was associated with the decomposition and depolymerization of pectin (~50%). The thermal degradation of the galacturonic acid chains caused the formation of various gaseous products [52,53]. The T_peak_ was determined in a range of 210–249 °C for all pectins. Moreover, in Figure 4a, it is possible to observe the TGA curve of the lignocellulose residue. The weight loss of the residue mainly occurred in the temperature range of 50–600 °C, which can be divided into three phases: 50–150 °C, 150–400 °C, and 400–600 °C [54]. Weight loss in the first phase (50–150 °C) was mainly caused by the evaporation of the absorbed water and the decomposition of low molecular weight polysaccharides. The second stage (150–400 °C) was mainly caused by the degradation of hemicellulose, lignin, pectin, etc. Third stage (400–600 °C) was slow, probably as a result of the decomposition of complex lignin polymeric compounds [55].

As regards the DTG curves of the pectins (Figure 4b), a mass variation was observed in a very small and wide peak, at a temperature of around 100 °C, corresponding to the evaporation of water [56]. However, it was possible to note three flex points in the DTG curve of the PEC-6h-3%. The first flex point occurs in an interval between 150–200 °C probably due to a polyphenol and sugar fraction still present in the pectin after washing with alcohols. The second flex point occurs in an interval between 210 and 249 °C due to the thermal degradation of the pectin. The third flex point, wider and less clear, can be observed at 300 °C and is probably due to the thermal degradation of the residual hemicellulose present in the pectin [57].

In DTG curves of commercial citrus PEC and purified PEC, one can note the presence of the peak related to bioactive molecules and the lignocellulose fraction. This confirms that the purification treatment of extracted pectin is necessary in order to obtain a pectin with a similar structure to that of commercial citrus pectin. In Figure 4b, the DTG curve of the pectin residue is shown. Moreover, in this case, a mass variation was observed in the DTG curve of the residue, evidenced by a very small and wide peak at a temperature of around 100 °C, corresponding to the loss of water. In addition, two flex points were observed in the DTG curves. A first peak, more crowded and less clear, occurred at around 250 °C due to the thermal degradation of the hemicellulose fraction in the residues [58].

Finally, it was possible to note a clear and wide peak in a range between 300 and 400 °C, caused by the thermal degradation of the lignocellulose fraction residue.

### 3.4. Mechanical Tests: Tensile Test and Puncture Test

The Mechanical properties of the pectin film and biocomposites are summarized in Table 4 and Table 5. Young’s modulus (E) specifies the stiffness or rigidity of the film, tensile strength (σ) indicates the tensile strength of the film up to breaking, and elongation at break (ε) describes the flexibility or extensibility of the films up to breaking. The results, reported in Table 4, show that the tensile strength and Young’s modulus values of the purified pectin film are higher than those of the pectin film (PEC) obtained from the liquid extract, due to the higher molecular rigidity [27].

However, the PEC film demonstrated a higher value of elongation at breaking as compared to the purified pectin film, due to the presence of bioactive molecules (flavonoids, flavanones, anthocyanins, phenolic acids, etc.) and essential oils that act as plasticizers, which are able to increase polysaccharide macromolecular chain mobility and caused a sharp decrease in the E and σ. Tensile strength and elongation at breaking were usually related to the network of the film microstructure and the intermolecular force between them [59].

The results of the puncture test for the PEC, PEC-15, PEC-25, and PEC-35 biocomposites are reported in Table 5. From the data analysis, it was possible to observe an increase in the resistance to breaking when lignocellulose residue (filler) was added in an amount equal to 0.25 g [60]. The force to breaking of the films depended on the thickness and component ratio of the films. The thicker the film and the higher the concentration of filler, the more resistant; thus, the greater the force at breaking point. However, an increase in filler content causes a slight decrease in displacement at breaking point. The deformation at breaking point is connected to the elasticity of films [61].

This outcome could be due to the increase in defects (i.e., kinks), typically an indicator of inhomogeneity and a random dispersion filler acting as points of stress concentration. On the other hand, as the amount of filler increased, it is possible to see a decrease in the displacement at breaking point for the PEC-25 and PEC-35 samples, typical of a more stiff and less elastic material, as show in Table 5.

### 3.5. Scanning Electron Microscope (SEM)

**Figure 6:**
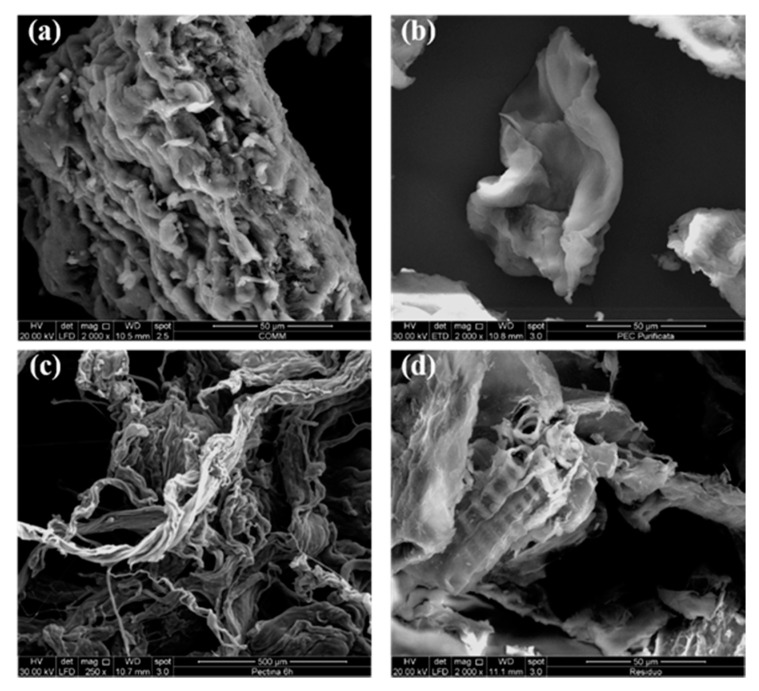
SEM micrographs of commercial pectin (**a**), purified PEC (**b**), PEC-6h-3% (**c**), and residue (**d**).

The SEM micrographs of PEC-6h-3%, purified pectin, commercial citrus pectin, and residue are shown in Figure 6a–c. From the SEM image of the commercial citrus pectin (Figure 6a), the material was verified to be fibrous with compact aggregates, i.e., it had an irregular morphology [62]. The PEC-6h-3% sample presented irregular intercellular spaces that were rough and uneven and compressed aspects (Figure 6c). In particular, it is possible to observe a few structural changes due to the treatment with acetic acid and water; thus, increasing the irregular porous surface structure and small particle size [63].

From the analysis of SEM picture of the purified pectin (Figure 6b), it is noted that the morphological structure is similar to that of commercial citrus pectin. On the other hand, comparing the PEC-6h-3% and purified pectin micrographs, a notable difference due to a presence of lignocellulose residues on the PEC-6h-3% surface can be seen. Thus, it is possible to conclude that the purification and centrifugation treatments allow large fat-soluble and lignocellulose residue fractions to be removed, causing evident morphological changes [64].

Finally, in Figure 6d, the micrograph of the residue is shown. In the residue, compact and joined fibrous cellulose structures were observed, showing some alignment. Each elementary fiber also had a compact structure; exhibiting an alignment with some other nonfibrous components on the residual surface (Figure 6d) [65].

The effect of the acid treatment, which occurred during the extraction of pectin, leads to a greater presence of coarse and disordered fibers with clear cusps (spool) for the longer extraction times. As regards the residue obtained after 6 h, it can be clearly seen in Figure 6d that the fiber bundles contain a large number of fiber cells aligned and delimited by lignin and hemicellulose [66]. Moreover, in the Figure 6d, we can see a cellular network due caused by the presence of interwoven and jagged microfibrils and to the treatment with acetic acid, which has smoothed the fiber surface [67].

**Figure 7:**
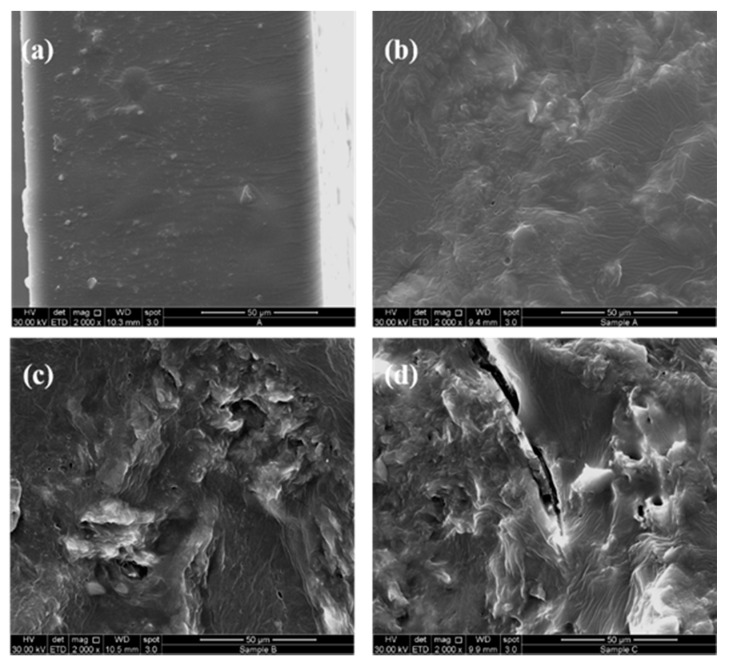
SEM micrographs of pectin film (**a**) and PEC-15 (**b**), PEC-25 (**c**), and PEC-35 (**d**) biocomposites.

The cross-sectional images of the biocomposites are shown in Figure 7a–d; micrographs of the PEC and PEC-15 samples with a homogeneous surface are shown in Figure 7a,b. In particular, it is possible to observe that the lignocellulose residue solid, in the PEC-15, is completely covered by a polymeric matrix, allowing for a good distribution and interfacial adhesion [68]. PEC-25 and PEC-35 biocomposites demonstrated (Figure 7c,d) an intense formation of defects (i.e., kinks) on their surface, and in the cross-section, due to the presence higher amounts of residue. This confirms the observation made from the mechanical properties, as discussed above. Thus, the filler (residue) causes a decrease in the displacement at the breaking point, resulting in breakage of the PEC-25 and PEC-35 samples and the formation of voids as a result of the removal of filler particles [69].

For this reason, from Figure 7c,d, it is possible to note the presence of rough and smoothed zones typical of a material with poor interfacial adhesion between the polymeric matrix and filler.

## 4. Conclusions

In this paper, we present our study on the optimization of the extraction conditions of citrus pomace using a combination of time and the concentration of mild organic solvents, such as acetic acid, to recover high M_w_ pectin. Full characterization of the obtained pectin and other organic components was carried out. The current global trend towards the efficient use of natural resources promotes sustainable agricultural production and the transformation of agricultural waste into high value-added products. Our preliminary study shows that the pomace itself and the biopolymers, such as pectin and cellulose, that can be obtained from this pomace have the potential to be used in the development of biocomposite membranes from aqueous acidic solutions, which can be utilized as agricultural mulching films. Finally, the bioactive molecules extracted from citrus pomace are currently the subject of an investigation related to their specific composition and functional activity. On the basis of the results, a biopolymer matrix will be assembled with the aim of producing bioactive food packaging materials. These studies will be the subject of a forthcoming paper.

## Figures and Tables

**Table 1:** Pectin-based film and biocomposite compositions and their identification codes.

Identification Code	Pectin (PEC) (g)	Residue (g)
Purified PEC	1	-
PEC	1	-
PEC-15	1	0.15
PEC-25	1	0.25
PEC-35	1	0.35

**Table 2:** Percentage of yield and degree of methoxylation (DM) of pectin as a function of time (h) and acetic acid concentration (%).

Data/Samples	PEC-2h-9%	PEC-4h-9%	PEC-6h-9%	PEC-6h-3%
Yield (%)	16.4 ± 3.4	20.3 ± 2.2	23.7 ± 1.8	24.1 ± 2.6
DM (%)	70.6 ± 3.2	58.3 ± 3.4	57.8 ± 3.0	70.3 ± 3.3

**Table 3:** Molecular weight parameters and intrinsic viscosity of extracted pectins vs. different processing times and acetic acid concentrations.

Samples	M_n_ (Da)	M_w_ (Da)	IV
PEC-2h-9%	55,503	140,719	1.53
PEC-4h-9%	39,320	116,889	0.96
PEC-6h-9%	25,443	91,293	0.57
PEC-6h-3%	12,6486	308,847	1.69

**Table 4:** Mechanical properties (tensile test) of Purified PEC and PEC films.

Data/Samples	Young Modulus (E) (MPa)	Tensile Strength (σ) (MPa)	Elongation at Break (ε) (%)
Purified PEC film	4045.0 ± 1051	22.0 ± 22	0.7 ± 0.4
PEC film	46.0 ± 33	3.2 ± 2	31.2 ± 9.3

**Table 5:** Mechanical properties (puncture test) of the pectin film and biocomposites.

Data/Samples	Force to Break (N)	Displacement (mm)
PEC	5.3 ± 1.8	7.8 ± 1.2
PEC-15	19.9 ± 7.4	8.3 ± 0.7
PEC-25	27.6 ± 7.9	6.9 ± 3.3
PEC-35	3.7 ± 1.2	4.6 ± 0.5

## Data Availability

Not applicable.
